# Efficacy of Regional Anesthesia in Reducing Perioperative Pain and Delirium in Elderly Patients Undergoing Hip Fracture Surgery: An Umbrella Review

**DOI:** 10.7759/cureus.93638

**Published:** 2025-10-01

**Authors:** Navod Jayasuriya, Muaz Ali, Abdaal Munir, Jamal Montaser, Srihas Tumu, Venkata Yashashwini Maram Reddy, Lubna Mohammed

**Affiliations:** 1 Anesthesiology and Perioperative Medicine, Faculty of Medicine, University of Ruhuna, Galle, LKA; 2 Medicine, Rawalpindi Medical University, Rawalpindi, PAK; 3 Surgery, College of Physicians and Surgeons of Pakistan, Islamabad, PAK; 4 General Surgery, Wirral University Teaching Hospital NHS Foundation Trust, Birkenhead, GBR; 5 Psychiatry and Behavioral Sciences, California Institute of Behavioral Neurosciences and Psychology, Fairfield, USA; 6 Internal Medicine, Guntur Medical College, Guntur, IND; 7 Principles and Practice of Clinical Research, Harvard T.H. Chan School of Public Health, Boston, USA

**Keywords:** epidural anesthesia, femoral fracture, hip fracture, neuraxial anesthesia, orthopaedic surgery, regional anesthesia, spinal anesthesia

## Abstract

The incidence of hip fractures is a significant health concern, especially among the elderly, who are an important population to consider while doing anesthesia. Although clinical guidelines support regional anesthesia (RA) for pain management, its consistent use in hip fracture surgery remains limited. This umbrella review evaluates the efficacy of RA in reducing perioperative pain and the incidence of delirium in hip fracture patients. A comprehensive search was conducted according to Preferred Reporting Items for Systematic Reviews and Meta-Analyses (PRISMA) 2020 guidelines across five databases (PubMed, PubMed Central, Google Scholar, ScienceDirect, and Cochrane Library) for studies published between January 1, 2015, and January 1, 2025. Of 1727 articles identified, a total of 16 systematic reviews and/or meta-analyses met the inclusion criteria after rigorous screening with the help of Microsoft Excel (Microsoft® Corp., Redmond, WA, USA) and a quality assessment according to the AMSTAR (A MeaSurement Tool to Assess Systematic Reviews) score system. Findings indicate that RA, particularly spinal anesthesia combined with femoral and fascia iliaca compartment blocks (FICBs), offers significant benefits over general anesthesia, including reduced opioid use, lower pain scores, and decreased risk of postoperative delirium. These results support RA as a preferred approach for improved outcomes, such as shorter hospital stays and enhanced postoperative recovery in geriatric patients.

## Introduction and background

Hip fractures have emerged as a growing health problem, with the number of cases projected to reach 6.26 million annually by 2050 due to population aging [1]. Between 2000 and 2012, the Nationwide Inpatient Sample (NIS) reported 4.9 million hospitalizations for osteoporotic fractures among women aged 55 and older, including 2.6 million hip fractures [2]. In Europe and the USA, hip fracture mortality ranges from 4% to 12% within the first month, and it can reach 35% within a year, causing most patients to need hospitalization and surgery because of the high morbidity and mortality rates [3]. The global rise in hip fractures, driven by an aging population, poses significant challenges to both patients and healthcare systems [3]. Many fractures are complicated due to the age and underlying medical conditions of the patients [3].

Regional anesthesia (RA) blockade techniques are categorized into central neuraxial blocks (spinal, epidural, and combined spinal and epidural) and peripheral nerve blocks (PNBs) [4]. They temporarily block pain transmission and may also cause muscle motor blockades, depending on the type of local anesthesia and its concentration [2].

Central neuraxial block involves administering a local anesthetic solution near the spinal cord, with the spinal block delivering it into the cerebrospinal fluid and the epidural block into the epidural space [5]. PNBs can serve as an alternative to general anesthesia (GA) during surgery, as a better treatment for perioperative pain, or to reduce the need for systemic medications during GA [2]. Neuraxial or PNBs are given as a single injection or in incremental doses, with or without continuous infusion, to keep the patient conscious yet pain-free under RA [5]. Central neuraxial block is the most frequently used anesthetic method for hip fracture surgeries [6]. But patients with hip fractures endure severe pain, making it difficult to position them properly for a neuraxial blockade [6]. Femoral nerve block (FNB), fascia iliaca block (FICB), and three-in-one block were previously utilized for hip fractures, offering perioperative analgesia and helping to reduce postoperative opioid consumption [6].

It is recommended to start pain management before admitting the patient to the hospital to avoid any potential mental disorders and delirium because of the intense pain that usually occurs in 50%-70% of femur fracture cases within 24 hours [7]. Postoperative delirium (POD) is a common and serious complication in elderly patients with hip fractures [1,8]. Although GA has been linked to higher POD risk via effects on cerebral function and neuroinflammation, recent trials show no clear difference between general and spinal anesthesia (SA), warranting further study [1]. RA is preferred over opioids, which are linked to higher delirium risk and side effects [9]. Regional nerve block (RNB) is also associated with quicker pain relief, a lower risk of inadvertent intravascular injection, and reduced local anesthetic usage [10]. Neuraxial anesthesia is increasingly used as an alternative to GA due to its short-term benefits, such as potentially reduced risk of POD due to minimal or no intraoperative sedation [11]. Recent studies have focused on examining the potential of RA to prevent chronic postoperative pain [12].

The optimal anesthetic strategy for older patients undergoing hip fracture surgery is still debated despite these differences [13]. Though most anesthesiologists can perform nerve blockade, integrating it into routine care has been challenging, as many avoid it due to concerns that the time, effort, and supervision required may not justify the benefits [9]. The clinical practice guidelines from the American Academy of Orthopaedic Surgeons (AAOS) on managing hip fractures in the elderly recommend the use of RA to enhance preoperative pain control [14].

**Figure 1:**
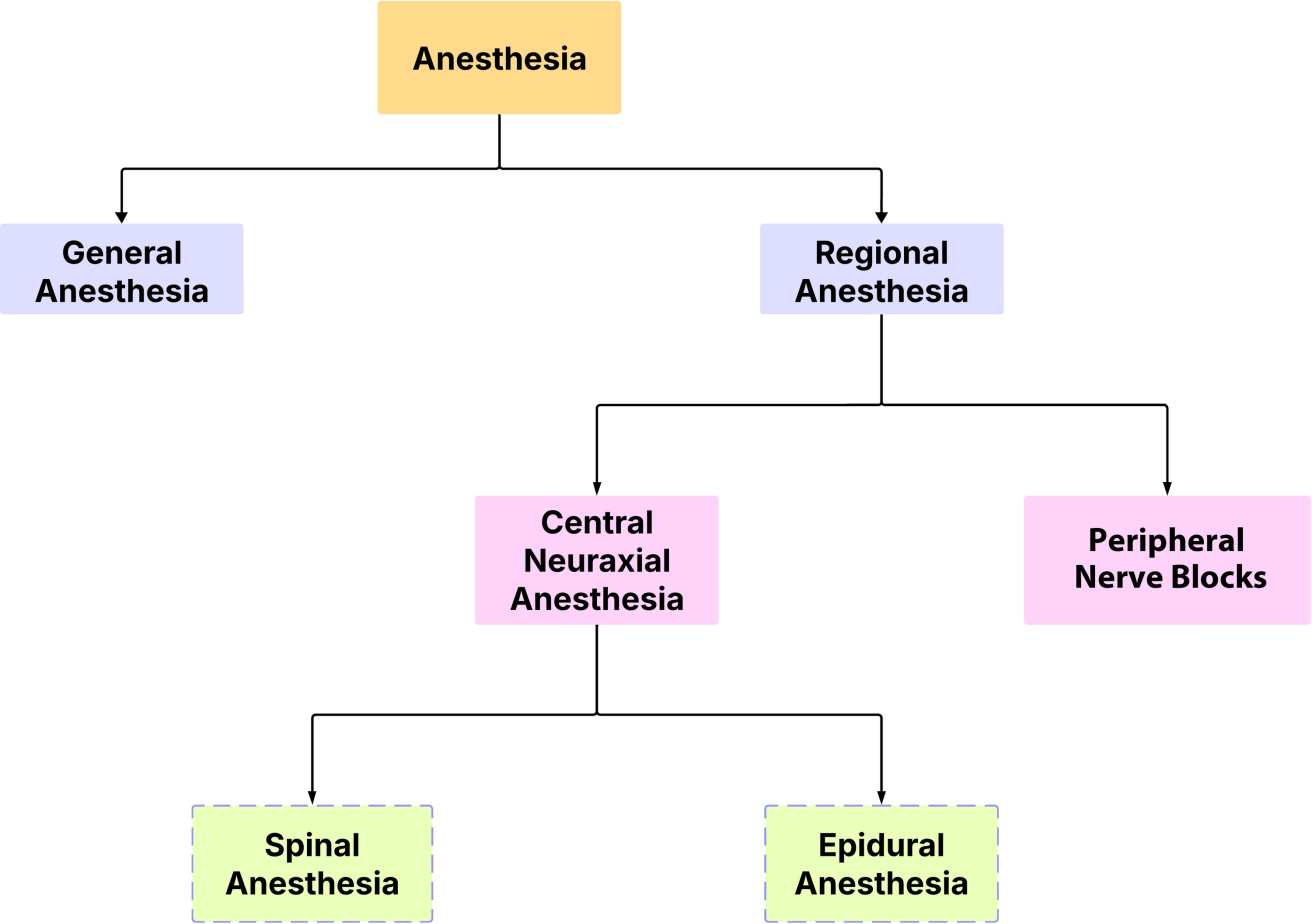
Flowchart of anesthesia technique classification Image Credit: Navod Jayasuriya

Although these procedures offer potential benefits and national guidelines advocate for their use, studies in the United Kingdom and Australia have shown that RNBs are not being consistently utilized [14]. Commonly used anesthetic techniques can be classified as general and regional, with RA including epidural and SA [15]. Figure 1 presents the general classification of anesthesia techniques commonly practiced by anesthesiologists in various procedures.

After studying this issue, we developed the research question, “How effective is RA in reducing perioperative pain and delirium in patients undergoing hip fracture surgery?”

Current evidence suggests that RA is becoming a safer and more effective method than GA for managing pain in elderly patients with hip fractures and complications like delirium. Though RA has many advantages in managing patients with hip fractures during surgery, it is still not widely practiced. The purpose of this study is to assess the effectiveness of RA compared to GA in lowering pain and delirium in hip fracture patients. This review aims to discuss effective strategies that help physicians improve pain management with better outcomes. Collectively, our findings will contribute to a better understanding of the role of RA in reducing perioperative complications and promoting its broader adoption in clinical practice.

## Review

Methods

This umbrella review adhered to the Preferred Reporting Items for Systematic Reviews and Meta-Analyses (PRISMA) 2020 guidelines [16]. Duplicate articles were removed, and the identification, screening, and organization of full-text articles were carried out manually based on inclusion and exclusion criteria with the assistance of Microsoft Excel (Microsoft® Corp., Redmond, WA, USA). To reduce the selection bias, blind assessments were performed, allowing several reviewers to independently classify studies without being influenced by each other’s decisions.

We performed a Corrected Covered Area (CCA) analysis on all 16 systematic and/or meta-analyses and identified 240 total primary study citations across the evidence base. This aggregation resulted in a CCA size of 158 unique primary studies. The calculated CCA percentage was 65.8%, indicating a moderate level of primary study overlap, which was considered to prevent a unit-of-analysis error.

Eligibility Criteria

This umbrella review included studies that met the following criteria: Only systematic reviews and/or meta-analyses that follow a consistent umbrella review design, written in English, conducted in any country, involving human participants (both male and female), and available as free full-text articles published between January 1, 2015, and January 1, 2025. Additional inclusion criteria were studies examining the effectiveness of RA in managing pain and delirium in patients undergoing hip fracture surgery. Exclusions included animal studies, book chapters, translated papers, conference papers, editorials, abstract-only papers, protocols, and grey literature. Further exclusion criteria were studies published before 2015, paid articles, studies in languages other than English, studies focused on the efficacy of RA in surgeries other than hip fracture surgery, and studies examining neurocognitive outcomes other than delirium. The decision to exclude studies published before 2015 was made to ensure the review remained focused on recent evidence and to prevent the study from becoming overly extensive by including older literature.

Databases and Search Strategy

We searched for relevant articles on PubMed (MEDLINE), PubMed Central, Google Scholar, ScienceDirect, and the Cochrane Library. Keywords and Medical Subject Headings (MeSH) terms were utilized to identify potentially relevant articles addressing the efficacy of RA in managing pain and delirium in patients undergoing hip fracture surgery. The search included keywords such as RA, epidural anesthesia, SA, RNB, intrathecal anesthesia, hip fracture, hip surgery, femoral neck fracture, hip joint fracture, femoral head fracture, and trochanteric fracture. The Boolean method was applied to combine the keywords and MeSH terms for searching the various databases. The search strategies employed in this systematic review are detailed in Table 1.

**Table 1:** Overview of the search strategy employed in this umbrella review

Database	Search strategy	Filters applied	Results
PubMed	Regional anesthesia OR epidural anesthesia OR Neuraxial anesthesia OR Regional nerve block OR Spinal anesthesia OR intrathecal anesthesia OR ( "Anesthesia, Conduction/adverse effects"[Majr] OR "Anesthesia, Conduction/classification"[Majr] OR "Anesthesia, Conduction/economics"[Majr] OR "Anesthesia, Conduction/ethics"[Majr] OR "Anesthesia, Conduction/history"[Majr] OR "Anesthesia, Conduction/instrumentation"[Majr] OR "Anesthesia, Conduction/methods"[Majr] OR "Anesthesia, Conduction/mortality"[Majr] OR "Anesthesia, Conduction/nursing"[Majr] OR "Anesthesia, Conduction/psychology"[Majr] OR "Anesthesia, Conduction/standards"[Majr] OR "Anesthesia, Conduction/trends"[Majr] ) AND Hip Fracture OR Femoral neck fracture OR Fracture of the femur OR Femoral head fracture OR Hip joint fracture OR trochanteric fracture OR Upper femur fracture OR ( "Hip Fractures/cerebrospinal fluid"[Majr] OR "Hip Fractures/classification"[Majr] OR "Hip Fractures/complications"[Majr] OR "Hip Fractures/diagnosis"[Majr] OR "Hip Fractures/diet therapy"[Majr] OR "Hip Fractures/drug therapy"[Majr] OR "Hip Fractures/economics"[Majr] OR "Hip Fractures/epidemiology"[Majr] OR "Hip Fractures/ethnology"[Majr] OR "Hip Fractures/mortality"[Majr] OR "Hip Fractures/pathology"[Majr] OR "Hip Fractures/physiopathology"[Majr] OR "Hip Fractures/prevention and control"[Majr] OR "Hip Fractures/psychology"[Majr] OR "Hip Fractures/rehabilitation"[Majr] OR "Hip Fractures/surgery"[Majr] OR "Hip Fractures/therapy"[Majr] )	Last 10 years, systematic review, free full text, English, humans	523
PubMed Central	((((((((((("regional anesthesia"[Abstract] OR "spinal anesthesia"[Abstract]) OR "epidural anesthesia"[Abstract]) OR "regional nerve block"[Abstract]) OR "intrathecal anesthesia"[Abstract]) OR "neuraxial anesthesia"[Abstract]) AND "hip fracture"[Abstract]) OR "hip joint fracture"[Abstract]) OR "femoral neck fracture"[Abstract]) OR "femoral head fracture"[Abstract]) OR "fracture of the femur"[Abstract]) OR "trochanteric fracture"[Abstract]) OR "pelvic fracture"[Abstract] AND ("open access"[filter] AND ("2015/01/01"[PubDate] : "2025/01/01"[PubDate]))	Last 10 years, systematic reviews, open access	766
Google Scholar	(“regional anesthesia" OR “neuraxial anesthesia” OR "regional nerve block" OR "spinal anesthesia" OR "epidural anesthesia" OR "peripheral nerve block”) AND (“post-operative pain") AND ("femoral neck fracture" OR “hip joint fracture” OR "pelvic fracture" OR "hip fracture surgery") efficacy	10 years	395
ScienceDirect	(“regional anesthesia" OR "regional nerve block" OR "spinal anesthesia" OR "epidural anesthesia" OR "peripheral nerve block”) AND ("femoral neck fracture" OR “hip joint fracture” OR "pelvic fracture" OR "hip fracture surgery"). Keywords - regional anesthesia, regional nerve block, spinal anesthesia, epidural anesthesia, peripheral nerve block, femoral neck fracture, hip joint fracture, pelvic fracture, hip fracture surgery	10 years, review articles, English, open access and open archive	40
Cochrane	Keywords - regional anesthesia, spinal anesthesia, epidural anesthesia, regional nerve block, intrathecal anesthesia, hip fracture, hip surgery, femoral neck fracture, hip joint fracture, femoral head fracture, trochanteric fracture	Last 10 years, Cochrane reviews, English	3

Results

Study Selection and Quality Assessment

We identified 1727 articles through various search strategies across five databases, comprising 523 from PubMed (MEDLINE), 766 from PubMed Central, 395 from Google Scholar, 40 from ScienceDirect, and three from the Cochrane Library. After manually identifying and eliminating 103 duplicate articles, we were left with 1624 articles, which we carefully examined and evaluated for relevance using their abstracts and titles. A total of 1,490 articles that were deemed irrelevant were found and eliminated. Next, 134 articles were selected for full-text retrieval, while 116 articles were excluded as they failed to fulfill our inclusion/exclusion criteria. The remaining 18 publications were assessed for quality by JN and AM using study-specific methods. Studies with scores more than 70% were included in our analysis, which used a grading system specific to each evaluation tool. A summary of the quality evaluation and the tools used is presented in Table 2.

**Table 2:** Information on the quality assessment and the tools used to evaluate the final articles included in this review AMSTAR: A MeaSurement Tool to Assess Systematic Reviews

Quality assessment tool	Type of study	Total score	Accepted score (>70%)	Number of accepted studies
AMSTAR	Systematic review, meta-analysis	16	>12	Exsteen et al., 2022 [17], Hsu et al., 2019 [18], Wan et al., 2020 [19], Hartmann et al., 2017 [20], Slade et al., 2023 [21], Hu et al., 2024 [22], Mou et al., 2024 [23], Patel et al., 2018 [24], Zhou et al., 2023 [25], Kim et al., 2022 [26], Fanelli et al., 2022 [27], Van et al., 2017 [28], Chen et al., 2019 [29], Ma et al., 2023 [30], Chen et al., 2023 [31], Kunutsor et al. 2022 [32]

Two additional articles were excluded as they scored below 70% according to our criteria. As a result, 16 systematic review articles were included in the study.

AMSTAR (A MeaSurement Tool to Assess Systematic Reviews) is a 16-point tool used to evaluate the methodological quality of systematic reviews. A score above 12 is generally considered indicative of high-quality reviews. Table 2 presents the AMSTAR assessment of the selected studies, evaluating their methodological quality according to the 16-point scoring system.

**Figure 2:**
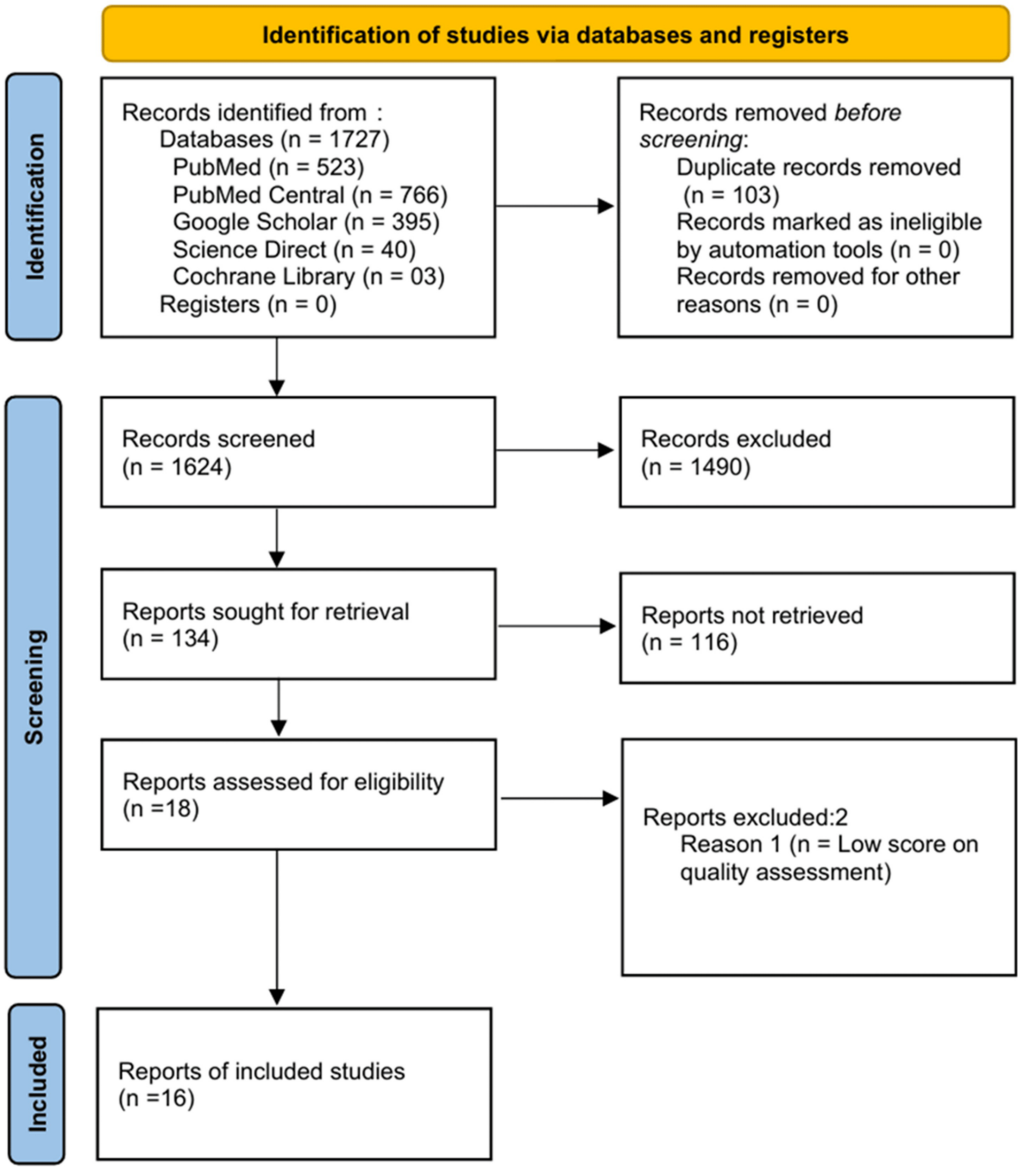
PRISMA flow chart illustrating the selection process of the included articles PRISMA: Preferred Reporting Items for Systematic Reviews and Meta-Analysis

The PRISMA flow chart, which outlines the identification, screening, eligibility, and inclusion process of the selected studies, is presented in Figure 2.

Citation Matrix and Overlap Analysis

The CCA analysis confirmed a moderate overlap (65.8%), with 48 unique primary studies included in two or more systematic reviews. The primary studies exhibiting the highest degree of redundancy (cited in three or more systematic reviews and/or meta-analyses) are detailed in Table 3.

**Table 3:** Highly overlapping primary studies (cited in greater than or equal to three systematic reviews and/or meta-analyses)

Primary study (first author, year)	Reviews cited	Total count
Urwin SC, 2000	[26,27,30,31]	4
Davis FM, 1987	[27,28,30,31]	4
Sorenson RM, 1992	[26,27,31]	3
Li T, 2022	[17,25,27]	3
Morrison RS, 2003	[17,19,23]	3
Jadad AR, 1996	[20,24,25]	3
Luger TJ, 2012	[17,23,24]	3
Neuman MD, 2018	[23,27,30]	3
Foss NB, 2007	[19,23,24]	3
Guay J, 2016	[25,27,28]	3
Messina ZJ, 2013	[28,30,31]	3
Parker MJ, 2015	[27,28,30]	3

Study Characteristics

Table 4 presents the characteristics of each selected study.

**Table 4:** Key characteristics of the studies included in this review RCT: randomized controlled trial; US: ultrasound; FNB: femoral nerve block; FICB: fascia iliaca compartment block; PNB: peripheral nerve block; RA: regional anesthesia; GA: general anesthesia; PENG: pericapsular nerve group; RNB: regional nerve block; POD: postoperative delirium; CAM: Confusion Assessment Method; DSM-IV: diagnostic and Statistical Manual of Mental Disorders, 4th edition; AKI: acute kidney injury; SA: spinal anesthesia; PONV: postoperative nausea and vomiting

Author and year	Study type	Inclusion and exclusion criteria	Sample size	Outcomes and key points
Exsteen et al., 2022 [17]	Systematic review	Inclusion Criteria: RCTs on adults (18+) undergoing proximal femur fracture surgery, comparing US-guided nerve blocks (FNB, FICB, 3-in-1 block) to traditional pain management. Exclusion Criteria: Quasi-randomized trials, observational studies, non-US-guided nerve blocks, and blocks performed during or after surgery.	976	Ultrasound-guided peripheral nerve blocks improved pain management, reduced opioid use, and decreased delirium and adverse events in hip fracture patients, with higher satisfaction, emphasizing the benefits of US-PNBs over conventional analgesia.
Hsu et al., 2019 [18]	Systematic review and meta-analysis	Inclusion Criteria: All published human RCTs or observational studies with a suitable control group. Exclusion Criteria: Case reports, case series, and abstracts.	584	FNB reduced pain more effectively than IV analgesics during spinal anesthesia, shortened procedure time, and improved satisfaction, with no significant hemodynamic impact, making it a safe choice for femur fracture patients.
Wan et al., 2020 [19]	Systematic review of randomized controlled trials	Inclusion Criteria: RCTs on FICB for perioperative pain in elderly hip fracture patients, published in English (2000-2020). Exclusion Criteria: Studies using FICB on non-geriatric or non-hip fracture patients.	2478	FICB provides effective pain relief, reduces opioid use, enhances spinal anesthesia, speeds recovery, and is safe, reliable, and easy, even in emergencies.
Hartmann et al., 2017 [20]	Systematic review	Inclusion Criteria: RCTs comparing FNB to fentanyl in femoral fracture patients of all ages and genders. Exclusion Criteria: Non-randomized studies or those not directly comparing the two interventions.	84	FNB outperformed fentanyl in pain prevention, analgesic reduction, and complication minimization during spinal anesthesia, improving efficacy and comfort. While it offers better pain management with fewer side effects, further studies are needed.
Slade et al., 2023 [21]	Systematic review and meta-analysis	Inclusion Criteria: Adults (GCS 14+) with femoral fractures in prehospital settings, receiving FICB or alternative analgesia. Includes observational studies or RCTs. Exclusion Criteria: Studies that do not meet the inclusion criteria or lack relevant outcome data.	340	FICBs reduced pain by 6.65 points, had an 89.3% success rate, minimal short-term adverse events, and high patient satisfaction. Simple and effective prehospital pain management.
Hu et al., 2024 [22]	Systematic review and meta-analysis of randomized controlled trials	Inclusion Criteria: RCTs on adults undergoing hip fracture surgery, comparing PENG block vs. sham/no block for analgesia, reporting pain, opioid use, nausea, dissatisfaction, and recovery. Exclusion Criteria: Studies involving combinations of the PENG block with other interventions.	831	The PENG block reduced opioid use, dynamic pain, and dissatisfaction, improving early recovery without affecting static pain or nausea. More research is needed due to limited, moderate-to-low-quality evidence.
Mou et al., 2024 [23]	Systematic review and meta-analysis	Inclusion Criteria: RCTs on PENG block vs. FICB for postoperative pain management in hip fracture surgery, assessing pain control, safety, recovery time, complications, and patient satisfaction. Exclusion Criteria: Studies involving patients under 18 years, non-hip fracture surgeries, or not evaluating PENG block/FICB with incomplete data, non-original research, or duplicates.	246	Both the PENG block and FICB effectively manage postoperative pain and reduce opioid use. PENG block improves mobility, reduces quadriceps weakness, and doesn’t increase PONV, enhancing recovery and function without significant publication bias.
Patel et al., 2018 [24]	Systematic review	Inclusion Criteria: Patients aged over 60 with fragility hip fractures undergoing regional vs. general anesthesia in randomized or non-randomized trials. Exclusion Criteria: Outdated anesthetic techniques, hip fractures with other multiple traumas, and uncontrolled studies.	104	The review found no strong evidence that anesthesia type affects postoperative delirium, mortality, or hospital stay. Regional anesthesia may offer quicker recovery, while GA is linked to more respiratory complications and hypotension.
Zhou et al., 2023 [25]	Systematic review and meta-analysis of randomized controlled trials	Inclusion Criteria: RCTs comparing RA vs. GA in older hip fracture surgery patients, focusing on delirium, mortality, and perioperative outcomes. Exclusion Criteria: observational studies, abstracts, reviews, and case reports.	3736	No significant difference in postoperative delirium or mortality between RA and GA. RA reduces operative time, blood loss, postoperative pain, hospital stay, and AKI risks.
Kim et al., 2022 [26]	Systematic review and meta-analysis of randomized controlled trials	Inclusion Criteria: RCTs comparing perioperative delirium between RNB and control groups in proximal femoral fractures (femoral neck, pertrochanteric). Exclusion Criteria: Non-RCTs, biomechanical studies, expert opinions, reviews, meta-analyses, conference abstracts, case reports, duplicate studies, or those with insufficient data.	941	RNB reduced delirium in elderly patients without preoperative cognitive impairment, but not in those with cognitive issues. No significant differences were found between block techniques.
Fanelli et al., 2022 [27]	Systematic review and meta-analysis	Inclusion Criteria: RCTs on adults (>18) comparing RA with systemic treatments, assessing POD using CAM or DSM-IV within 7 days post-surgery. Exclusion Criteria: Studies not screened for preoperative delirium, reporting outcomes beyond 3 months, focusing on cognitive dysfunction instead of POD, non-randomized, or lacking dichotomous outcomes.	3361	RA anesthesia during surgery did not significantly reduce the POD risk, but RA lowered it after surgery compared to systemic analgesia. For hip fractures, RA also reduces POD risk. Intraoperative RA alone was not effective in preventing POD, as other factors may contribute. RA post-surgery is an effective opioid-sparing strategy.
Van et al., 2017 [28]	Systematic review and meta-analysis	Inclusion criteria: Human studies (2016-2020) comparing general vs. neuraxial anesthesia for hip fractures, reporting 30-day mortality, in-hospital mortality, or length of stay. Includes prospective, randomized, and observational studies in any language. Exclusion Criteria: case series and systematic reviews, studies unrelated to anesthesia type, and studies without a clear definition of “local anesthesia.”	413,999	Neuraxial anesthesia reduced in-hospital mortality and length of stay but did not affect 30-day mortality. The study calls for larger trials to explore anesthesia types, complications, and mortality further.
Chen et al., 2019 [29]	Systematic review and meta-analysis	Inclusion Criteria: studies (2000-2018) evaluating perioperative outcomes of GA vs. RA in geriatric hip fracture surgery patients, focusing on 30-day mortality, in-hospital mortality, complications, hospital stay length, and readmission.	196,646	RA improves perioperative outcomes in geriatric hip fracture patients, reducing in-hospital mortality, acute respiratory failure risk, and hospital stay length. It also lowers readmission rates, but large RCTs are needed to confirm these findings.
Ma et al., 2023 [30]	Systematic review and meta-analysis	Inclusion Criteria: Studies (2002-2023) on perioperative outcomes of GA vs. RA in adults with hip fractures, including prospective and retrospective RCTs and observational studies, addressing hospital mortality, 30-day mortality, postoperative pneumonia, and delirium. Exclusion Criteria: Case reports, case series, systematic reviews, and meta-analyses.	363,470	RA reduced in-hospital mortality compared to GA. No difference in the reduction of postoperative pneumonia and POD between GA and RA.
Chen et al., 2023 [31]	Systematic review and meta-analysis	Inclusion Criteria: RCTs on adults (>18) with hip fractures undergoing surgery, comparing general vs. neuraxial anesthesia, reporting at least one quantitative outcome. Exclusion Criteria: Studies using RA other than neuraxial, without reported outcomes or patient group sizes; conferences; abstracts; animal/cadaveric studies; or non-clinical trials.	4802	GA increased the risk of postoperative AKI but showed no significant differences from neuraxial anesthesia in delirium, short-term mortality, cardiac infarction, acute heart failure, or pulmonary embolism. The study compared complications and mortality risks between GA and neuraxial anesthesia in hip fracture surgery.
Kunutsor et al., 2022 [32]	Systematic review and meta-analysis of randomized controlled trials	Inclusion Criteria: RCTs comparing spinal anesthesia vs. general anesthesia in hip fracture surgery, reporting at least one core outcome (e.g., mortality, delirium) or patient-important outcomes (e.g., quality of life, mobility). Exclusion Criteria: Non-randomized studies, studies using outdated anesthetic techniques, or studies involving patients with multiple surgeries or polytrauma.	3866	The study found no significant differences between SA and GA in most outcomes, except for a reduced risk of acute kidney injury with SA.

Discussion

Perioperative Pain Control of RA in Hip Fractures

Hip fractures are a significant health issue primarily affecting the elderly, resulting in severe pain during the perioperative period, with femoral neck fractures occurring at a rate of 9.5 to 18.9 per 100,000 people annually [17,18].

Current pain management involves paracetamol, opioids, nonsteroidal anti-inflammatory drugs (NSAIDs), anesthesia, and nerve blocks [19]. While opioids and NSAIDs are commonly used for pain relief, they may not address dynamic pain effectively, and they may also lead to side effects such as respiratory depression and delirium, especially in older adults, where these risks are increased due to impaired renal and hepatic function [19,20]. Regional anesthetic techniques provide better pain relief than oral or parenteral analgesia in the hospital and help decrease opioid use in patients with femoral fractures [21].

Around 98% of femur fractures require surgery, with SA being preferred due to its lower mortality risk compared to GA [18]. SA requires the patient to be positioned in either a lateral decubitus or sitting position, but this can be difficult due to the unavoidable movement of the fractures of the femur, which results in significant pain [19]. Femoral fractures cause intense pain because the periosteum has the lowest pain threshold of all deep somatic structures, highlighting the importance of pain management [18]. Administering fascia iliaca compartment block (FICB) before SA in geriatric hip fracture patients improves pain management, enhances SA performance, provides effective postoperative analgesia, and boosts care quality and efficacy [19].

Perioperative PNBs help reduce pain, with the emerging pericapsular nerve group (PENG) block offering a promising approach to selectively block the femoral, obturator, and accessory obturator nerves while preserving the motor function [17,20]. Ultrasound (US)-guided PNB is more precise than blind techniques and significantly reduces pain in hip fracture patients [17]. Recent evidence suggests that administering PNBs at the accident site by paramedics or nurses effectively relieves pain and results in higher patient satisfaction [17]. However, a significant limitation in PNB studies for hip fractures is the failure to assess block success, mainly because dementia hinders dermatome testing and fractures restrict myotome evaluation [17].

Peripheral nerve blockades, especially the FICB, are becoming an increasingly preferred method for effective pain relief [19]. This technique demonstrated higher success rates in blocking the femoral, lateral cutaneous, and obturator nerves compared to the femoral nerve or the “three-in-one block” [21]. FICB may offer superior and long-lasting pain relief for hip fractures compared to morphine, with a quick onset and lasting effectiveness for up to eight hours [19]. The "three-in-one" block and FICB are both single-injection methods designed to block the femoral, lateral cutaneous, and obturator nerves [19].

A comparison of FICB and FNB guided by a nerve stimulator in femoral neck fracture patients found that FNB offered superior pain relief and reduced morphine use compared to FICB [19]. An FNB is a regional anesthetic technique employed by emergency medicine doctors to deliver anesthesia and pain relief to the affected leg [20]. FNB can be performed using a nerve stimulator, fascia iliac block with large anesthetic volumes, the “three-in-one” to target femoral, obturator, and lateral cutaneous nerves, or US guidance to locate the nerve [20].

The PENG block (PENGB) is a relatively new technique that primarily targets the articular branches of the femoral and obturator nerves [23]. It is now emerging as a better alternative to FICB, providing efficient pain management without causing significant muscle blockade [23]. The advantage of PENGB is enhanced by its minimal impact on the strength of the quadriceps muscle, allowing the patients to walk after surgery [23]. Although PENGB and FICB offer similar pain relief and opioid reduction, FICB is a suitable alternative when PENGB resources or expertise are unavailable [23].

The evidence suggests that RA, especially the FICB, offers significant pain relief for hip fractures, reduces opioid consumption, and leads to better postoperative results. Although newer techniques such as PENGB are promising, FICB remains a reliable and accessible choice for geriatric patients to enhance pain management and recovery when performed properly.

Table 5 summarizes some of the studies on different analgesia techniques and their effect on pain relief and patient positioning.

**Table 5:** Comparison of studies on different analgesia methods for pain relief FNB: femoral nerve block; FICB: fascia iliaca compartment block; SA: spinal anesthesia

Study and year	Study type	Anesthesia method comparison and study population (n)	Outcome parameters	Key conclusions
Guay et al., 2019 [18]	Systematic review and meta-analysis	FNB (n=292); intravenous (IV) analgesic (IVA) (n=292)	Pain during movement was assessed 30 minutes before SA, along with the time needed for SA, hemodynamic changes, patient acceptance, and use of additional analgesics	FNB proved to be an effective and safe method for positioning femur fracture patients for spinal block, especially those receiving SA in the sitting position, compared to IVA.
Madabushi et al., 2016 [19]	Systematic review of randomized controlled trials	FICB (n=30); IV fentanyl (n=30)	Visual Analogue Scale (VAS) score, sitting angle, time to achieve SA, and positioning quality	Markedly enhanced sitting angle in the FICB group.
Yun et al., 2009 [19]	Systematic review of randomized controlled trials	FICB (n=20); IV alfentanil (n=20)	VAS score, patient satisfaction, time required for SA	Reduced mean VAS score during patient positioning, quicker time to achieve SA, and better patient acceptance.
Yamamoto et al., 2019 [19]	Systematic review of randomized controlled trials	FICB (n=25); IV acetaminophen (n=28)	VAS score, overall amount of rescue analgesics needed	FICB provided better postoperative pain relief during movement than IV acetaminophen, without raising the complication rate.
Dochez et al., 2014 [21]	Systematic review and meta-analysis	FICB (n=100)	Non-verbal pain score (NVPS)	The procedure is considered successful if the overall NVPS score drops by 4 points.

Reduction of Delirium Incidence With RA

Delirium is a common neuropsychiatric syndrome characterized by sudden changes in attention, awareness, and cognition, often fluctuating throughout the day [24]. Delirium, affecting about 61% of elderly hip fracture patients perioperatively, is a common complication of hip fracture repair that significantly raises mortality, morbidity, cognitive decline, and healthcare costs [25,26].

Risk factors for delirium include preoperative cognitive disorders, psychiatric illness, aging, reduced functional status, and severe postoperative pain [26]. Several studies have explored the impact of perioperative pain management, a key factor, in reducing POD following hip fractures [26].

Opioids can cause delirium, with acute pain triggering it, prompting the use of RNBs like FICB and FNB as effective alternatives in hip fractures [24,26]. Although there is no specific treatment for delirium, careful perioperative management can help reduce its incidence and severity [24]. RNBs have proven effective in managing hip fracture pain with minimal systemic toxicity and are expected to reduce delirium by controlling pain, a key risk factor [26].

A key finding from the present evidence is that RNB reduces delirium after hip fracture surgery in patients without cognitive impairment but has no effect on those with cognitive impairment [26]. It has been observed that cognitively intact hip fracture patients with untreated pain are nine times more likely to develop delirium than those with effective pain management [19].

FICB didn’t show a prophylactic effect in high-risk patients, but it significantly reduced delirium in intermediate-risk patients, indicating its potential benefit for perioperative delirium in this group [19]. Ultrasonography-based neurostimulator techniques are the standard of care in RA, with studies showing that ultrasonography-based FICB provides greater analgesia than FICB without US guidance [26]. A possible explanation for the reduced risk of delirium following FICB is the lower use of supplementary analgesics [19]. So, extending RA into the postoperative period may enhance its benefits on POD by reducing the need for opioids and sedatives during surgery [27].

Guideline committees recommend RA, if not contraindicated, as it avoids GA and opiates, which are linked to POD [24]. European guidelines also suggest RA and opioid-sparing approaches to prevent POD, though evidence supporting this is of low quality [27].

In summary, RNBs, especially FICB, effectively reduce POD in hip fracture patients without cognitive impairment by managing pain and reducing opioid use. However, their efficacy may be limited to high-risk or cognitively impaired patients, suggesting a need for future research.

**Figure 3:**
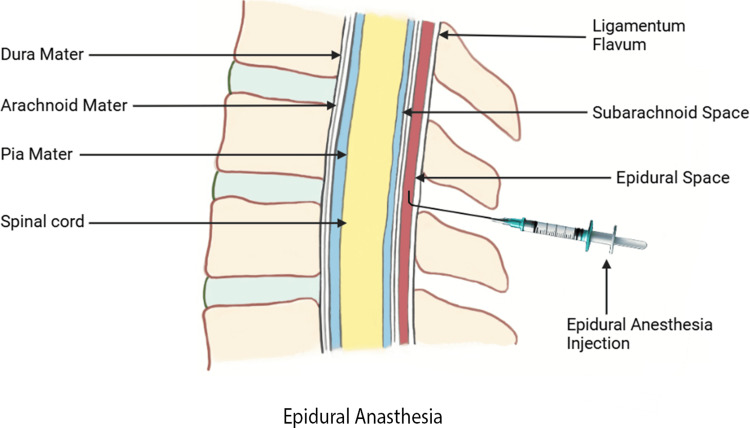
Illustration of epidural anesthesia administration technique Image Credit: Navod Jayasuriya

**Figure 4:**
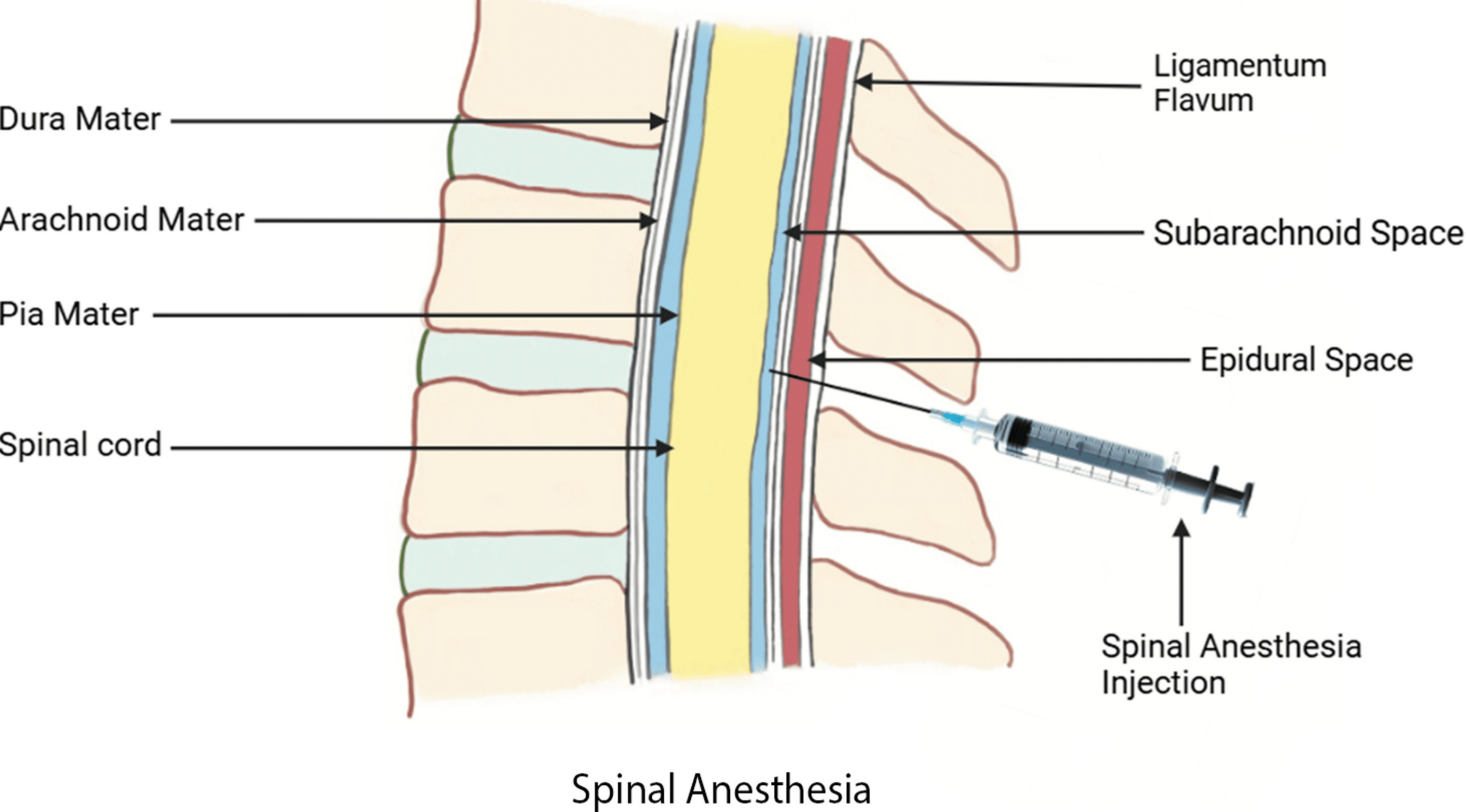
Illustration of spinal anesthesia administration technique Image Credit: Navod Jayasuriya

Figures 3-4 illustrate the two methods of administering epidural and SA, respectively.

Comparison of RA Versus GA in Hip Fracture Surgery

Anesthesia can generally be categorized into GA and RA [24]. Regional neuraxial block and GA are commonly used for hip fracture surgery, but to date, there is no consensus on which technique is optimal [25,28].

Evidence suggests that GA is linked to higher POD, longer perioperative stays, and mortality, with patients undergoing hip fracture surgery experiencing longer operative times, greater blood loss, higher incidence of acute kidney injury, more pulmonary and cardiovascular complications, and higher pain scores compared to those receiving RA; however, GA may have a lower risk of certain complications [24,25,28,29].

Ma et al. found that in hip fracture surgery, acute respiratory failure and stroke may be risk factors for in-hospital mortality in elderly patients under GA compared to RA, with patients treated in regional hospitals having a higher likelihood of experiencing postoperative stroke after receiving GA [30]. Some results showed no difference in 30-day mortality between GA and RA groups, but RA reduced mortality, hospital stay, and the incidence of myocardial infarction and respiratory failure [30].

When comparing RA and GA, myocardial infarction and thromboembolic events were the most frequently reported cardiovascular complications, with evidence indicating higher incidences of thromboembolic events and hypotension in hip fracture patients receiving GA [24]. Hypotension, leading to hypoperfusion and organ damage, is linked to increased mortality in hip fracture patients, particularly during GA, where excessive anesthesia depth and perioperative hypotension further elevate the risk [24,25]. However, RA does not eliminate the risk of perioperative hypotension and sedation [24]. Intravenous and inhaled anesthetics in GA, like sevoflurane, may raise the risk of delirium by introducing or worsening neuroinflammation [31]. GA may increase the risk of postoperative complications, including pneumonia, due to intubation and anesthetic use [31].

RA may offer benefits such as faster bowel recovery, a lower inflammatory response, reduced pulmonary dysfunction, and early post-anesthetic mobilization, which potentially improve cognitive function [24,27]. Postoperative pain scores, assessed with the Visual Analogue Scale (VAS), ranging from 0 to 10, were lower in RA patients, who also had shorter hospital stays (LOS) than GA patients, with acute pain being more intense in GA patients, particularly in hip fracture surgeries [25]. Postoperative nausea and vomiting (PONV) were considerably lower in the RA group compared to the GA group, possibly due to improved postoperative pain management and decreased opioid use in the RA group [25].

Some experimental evidence indicated a significantly higher risk of postoperative acute kidney injury (AKI) in patients receiving GA, while SA decreased the likelihood of AKI [31,32]. Research continues to investigate whether GA or neuraxial anesthesia is more effective at reducing postoperative complications, although some uncertainties about the two techniques remain [31,32].

FICB is used for lower extremity surgeries, lumbar plexus inflammation, cancer pain, and acute pain from fractures, trauma, or burns, but is contraindicated in patients with injection site infections, a history of femoral bypass surgery, coagulopathy, or those taking antithrombotic drugs [19]. FNB, a safe, simple, and easy-to-learn technique administered via the landmark method or US guidance, significantly lowers pain scores and reduces the time to perform SA, achieving greater reductions than IVA, particularly in the sitting position [18]. Evidence indicates that both patients and anesthesiologists favor FNB over IVA for pain relief and analgesia [18].

A key objective of this systematic review is to determine the most effective anesthesia technique for patients undergoing hip fracture surgery. This evidence suggests that RA is more beneficial than GA in reducing delirium, pain, length of hospital stays, and cardiovascular complications. However, as there may be situations where GA is still beneficial, further research is needed to analyze the best anesthetic technique for minimizing the risks and complications in elderly patients with hip fracture surgery.

Outcomes of RA During and After the Surgery

In managing patients with hip fractures, the choice of anesthesia is an important part of the multidisciplinary treatment approach, as it influences the risks of perioperative as well as long-term complications [29,31]. Recent studies show that hip fracture patients who receive GA have a higher risk of hospital re-admission, whereas those who receive neuraxial anesthesia have a significantly shorter hospital stay [28,29].

Effective pain management promotes early mobilization, reducing the risk of deep vein thrombosis (DVT), pulmonary embolism (PE), chronic pain, and psychological distress like anxiety and depression [23]. Additionally, evidence shows inconsistencies between 30-day mortality and in-hospital mortality, implying that anesthesia techniques have a more significant effect on short-term outcomes than on long-term outcomes [29].

RA carries potential risks, including anesthetic toxicity, nerve injury (temporary or permanent), allergic reactions, infection, and bleeding [21]. Unsurprisingly, the standard care group in randomized controlled trials experienced significantly more adverse outcomes, primarily respiratory depression due to opioid use [21]. Older patients face a higher risk of postoperative complications due to age-related decline, comorbidities, and polypharmacy [24]. Some evidence suggests the patient had a brief episode of hypertension and tachycardia lasting around 10 minutes after the block, with the symptoms resolved on their own within five minutes, without any lasting effects [21].

Although FICB is effective for postoperative pain relief, it has drawbacks, such as the risk of local anesthesia systemic toxicity (LAST), inconsistent block effects due to anatomical differences, potential nerve injury from needle trauma, and the potential for lower limb motor weakness [23]. Interestingly, a higher incidence of POD was observed in the FICB group, suggesting that factors beyond pain may contribute to delirium [19].

PENGB provides effective hip pain relief without notable motor impairment, whereas FICB may cause motor weakness that can delay postoperative recovery [23]. By reducing quadriceps weakness, PENGB supports essential muscle strength for activities like walking and standing while promoting early mobilization, which is key to recovery and preventing complications like DVT and PE [23]. Consequently, PENGB could reduce the length of hospital stays and accelerate recovery, thereby improving patient outcomes and lowering healthcare costs [23].

Despite efforts to optimize perioperative care, 30-day mortality in geriatric hip fracture patients is around 14%, with one-year mortality ranging from 17% to 37%, and 20% facing severe complications, underscoring the need for improved anesthesia care to enhance outcomes for these high-risk patients [29]. Currently, no definitive conclusions can be made regarding the impact of RA on perioperative outcomes [29].

In conclusion, anesthesia choice in hip fracture surgery affects perioperative outcomes, with RA offering benefits over GA, such as fewer readmissions and improved short-term results; however, it carries risks like nerve injury and anesthetic toxicity.

Study Limitations

This umbrella review has some limitations that should be acknowledged to provide a more balanced perspective and minimize any potential bias in the interpretation of the findings. First, it was limited to systematic reviews and/or meta-analyses, and key databases such as Embase were omitted. In addition, only free full-text articles published in English within the past 10 years were included, which may have excluded relevant studies in other languages or older publications with valuable insights. Drawing firm conclusions was also challenging due to variations in study designs, populations, and anesthetic methods among the included studies. Furthermore, heterogeneity in reported results may have been influenced by differences in outcomes, sample sizes, follow-up periods, and methodological quality. Finally, a comprehensive assessment of the long-term efficacy of RA in hip fracture patients was limited by the scarcity of available evidence.

## Conclusions

This research has studied how RA may help elderly patients with hip fractures control pain and delirium. Effective regional anesthetic procedures may provide the best perioperative care for patients undergoing hip fracture surgery with minimal problems. Also, using SA combined with PNBs like FICB can yield better advantages. It has been highlighted that those patients with hip fractures who are more likely to have complications need customized pain management. Our study emphasizes how important RA is in obtaining better outcomes like shorter hospital stays, reduced postoperative complications, and increased mobility after surgery as well. But it also acknowledges the possible risks of RA, including anesthetic toxicity and nerve damage that can occur during the procedures. RNBs, especially FICBs, reduce POD in hip fracture patients without cognitive impairment by providing effective pain control and limiting opioid use. Their benefit, however, appears limited in cognitively impaired or high-risk patients. Extending RA postoperatively may further decrease delirium risk by reducing opioid and sedative needs. Guidelines recommend RA as an opioid-sparing strategy, though evidence quality remains low, underscoring the need for further research.

Future research should focus on improving anesthetic procedures, investigating more effective anesthetic techniques such as PENGB, and assessing how that anesthesia affects patients’ mortality and recovery after surgery. This umbrella review adds to the evidence indicating that RA is an effective option, especially for elderly patients undergoing hip fracture surgery, and its potential impact on patient care and outcomes.
